# Etoricoxib–NLC Mitigates Radiation-Induced Ovarian Damage in Rats: Insights into Pro-Inflammatory Cytokines, Antioxidant Activity, and Hormonal Responses

**DOI:** 10.3390/biom15010012

**Published:** 2024-12-25

**Authors:** Sahar Khateeb

**Affiliations:** 1 Department of Biochemistry, Faculty of Science, University of Tabuk, Tabuk 71491, Saudi Arabia; skhateeb@ut.edu.sa; 2 Biochemistry Division, Department of Chemistry, Faculty of Science, Fayoum University, Fayoum P.O. Box 63514, Egypt; sms07@fayoum.edu.eg

**Keywords:** radiotherapy, ovary, etoricoxib, nanostructured lipid carriers, PI3K/Akt, *Nrf2*, *TGF-β*

## Abstract

Radiotherapy is a critical treatment for cancer but poses significant risks to ovarian tissue, particularly in young females, leading to premature ovarian failure (POF). This study examines the therapeutic potential of etoricoxib nanostructured lipid carriers (ETO-NLC) in mitigating radiation-induced ovarian damage in female *Wistar* rats. Twenty-four female rats were randomly assigned to four groups: a control group receiving normal saline, a group exposed to a single dose of whole-body gamma radiation (6 Gy), a group treated with etoricoxib (10 mg/kg) post-radiation, and a group treated with ETO-NLC for 14 days following radiation. Histopathological evaluations and oxidative stress biomarker assessments were conducted, including ELISAs for reactive oxygen species (ROS), pro-inflammatory cytokines (IL-1β, TNF-α), and signaling molecules (PI3K, AKT, P38MAPK, AMH). Serum levels of estrogen, FSH, and LH were measured, and gene expression analysis for *TGF-β* and *Nrf2* was performed using qRT-PCR. The findings indicate that ETO-NLC has the potential to ameliorate the harmful effects of ovarian damage induced by γ-radiation. These therapeutic effects were achieved through the modulation of oxidative stress, inflammation, augmentation of antioxidant defenses (including *Nrf2* activation), support for cell survival pathways (via PI3K/Akt signaling), regulation of MAPK, mitigation of fibrosis (*TGF-β*), and preservation of ovarian reserve (as evidenced by AMH, FSH/LH, and estrogen levels). ETO-NLC shows promise as an effective strategy for attenuating radiation-induced ovarian damage, highlighting the need for further research to enhance therapeutic interventions aimed at preserving ovarian function during cancer treatment.

## 1. Introduction

A new field of study that has significant clinical utility in the treatment of young cancer patients is fertility preservation. Although the underlying cause of premature ovarian failure (POF) is complicated and still unknown, one risk factor for POF is radiation therapy (RI) [1]. Various cancer types frequently use ionizing radiation (IR) as a cytotoxic treatment [2], but it often damages healthy tissues, diminishing its capacity to fight malignancies. Furthermore, the ovaries are susceptible to significant exposure in cases of cancer treatment that involves abdominal, pelvic, or total body irradiation (TBI). In addition, treatment of lymphomas with TBI and co-irradiation of nearby solid tumors may cause the ovaries to become less functional, lead to premature ovarian insufficiency, and induce menopause [3]. Additionally, Kim et al. [4] showed that radiation can harm prepubescent girls and young women’s ovarian tissue.

RT causes water radiolysis that generates reactive oxygen species (ROS) in cancer cells, causing oxidative stress (OS) and a reduction in antioxidant defense systems that may also impact healthy normal tissues, such as the ovaries [5]. He et al. [6] further revealed that radiation-induced ovarian injuries result in oxidative damage and inflammatory injuries, downregulate PI3K and AKT expression, and induce ovarian follicular atresia. Nuclear factor erythroid 2-related factor 2 (*Nrf2*), a transcription factor, controls the expression of genes involved in the OS response, which in turn controls the cellular defense against oxidative assaults. Activation of *Nrf2* makes cells resistant to inflammation. Numerous other cellular functions are mediated by *Nrf2*, in addition to antioxidant responses [7]. Additionally, transforming growth factor-beta (*TGF-β*) is one of the biological signaling pathways that are unavoidably impacted when both healthy and malignant tissue are exposed to radiation. The *TGF-β* family consists of multifunctional cytokines with potent effects on numerous cellular functions. According to Barcellos-Hoff and Dix [8], ROS-mediated *TGF-β* activation predominates in tissues exposed to IR. Also, RT-induced granulosa cell death has recently been linked to the mitogen-activated protein kinase (MAPK) signaling pathway [9]. Therefore, it is imperative to implement strategies to mitigate and treat radiation damage, thereby protecting the ovaries.

Nonsteroidal anti-inflammatory drugs (NSAIDs) are one of several pharmacological classes that have been investigated as potential compounds that might decrease the negative effects of radiation [10]. The NSAID etoricoxib (ETO) is a member of the class of selective cyclooxygenase-2 (COX-2) inhibitors. ETO is used to alleviate pain and inflammation and is frequently prescribed for RT-assisted cancer treatment [11,12]. Nonetheless, it has poor solubility and can have negative effects when taken orally [13]. Consequently, efforts must be made to develop safer and more efficient chemicals for the treatment.

Pharmaceutical nanotechnology was used to enhance therapeutic delivery properties [14]. Nanostructured lipid carriers (NLCs) are gaining interest due to their effective drug delivery and encapsulation capabilities, which enhance the oral bioavailability of medications that are poorly water-soluble [15]. According to Bagherpour et al. [16], nano-encapsulation, which encloses the bioactive chemical within a carrier, can control the release of the bioactive molecule to enhance its solubility, resistance to oxidation, and bioavailability. The aim of the present study is to investigate the potential therapeutic effects of etoricoxib–NLC (ETO-NLC) against radiation-induced ovarian damage in female rats in comparison to conventional ETO therapy to provide new therapeutic options. This investigation includes assessing various parameters such as histological changes, oxidative stress markers [catalase (CAT), superoxide dismutase (SOD), malondialdehyde (MAD)], and the expression levels of key factors involved in inflammation (IL-1β, TNF-α), antioxidant response (*Nrf2*), cell survival (PI3K, AKT), fibrosis (*TGF-β*), and ovarian function (AMH, estrogen, FSH, LH) in response to radiation exposure. Additionally, this study aims to evaluate the levels of ROS in the ovarian tissue as a measure of oxidative damage.

## 2. Materials and Methods

### 2.1. Materials

Etoricoxib (Arcoxia) was bought from a general pharmacy and is manufactured by Merck & Co., Inc. in Hoddesdon, ENG, United Kingdom.Oleic acid and stearic acid were provided by Sigma-Aldrich Co., St. Louis, MO, USA. All other materials were of the analytical variety.

### 2.2. Preparation and Characterization of ETO-NLC

According to our previous research methodology [17], the ETO-NLC dispersions were created using the low-temperature melt emulsification solidification method developed by Sachan et al. [18]. The NLCs were characterized using a variety of methods, including zeta potential, polydispersity index distribution, particle size, scanning electron microscopy (SEM), X-ray diffraction (XRD), Fourier transform infrared spectroscopy (FTIR), the percentage of drug release of ETO-NLC, and the efficiency of drug entrapment and drug loading estimation.

### 2.3. Experimental Animal Groups

A total of 24 healthy female *Wistar albino* rats weighing 180–200 g were randomly assigned into four groups. For 14 days, Group 1 (control) received oral normal saline. Group 2 (γ-Rad) received a single dose of whole-body gamma radiation (6 Gy) and then continued to receive normal saline for the remainder of the trial. Group 3 (γ-Rad + ETO) received the same 6 Gy radiation as Group 2, and then, in accordance with Dave et al. [19], received treatment with ETO at a dose of 10 mg/kg body weight for 14 days. Lastly, Group 4 (γ-Rad + ETO-NLC) underwent the same 6 Gy radiation exposure as Group 2 and subsequently received ETO-NLC at a dose of 10 mg/kg body weight for 14 days. The selected dose and treatment duration were informed by similar studies conducted previously [20,21].

### 2.4. Sample Preparation

After 24 h following the last ETO or ETO-NLC treatment, the rats were anesthetized and humanely killed to enable the gathering of the samples. For hormone analysis, serum was separated by centrifugation for 15 min at 5000 rpm. Using a tissue homogenizer, the ovarian tissue (10% wt/v) from each rat group was promptly extracted and perfused with ice-cold PBS (0.01 M, pH = 7.4). The homogenates were centrifuged for 15 min at 4 °C at 5000 rpm. After centrifugation, the clear supernatant was used to evaluate biochemical parameters. The leftover ovarian tissue, stored in 10% formalin, was used for histopathological examinations.

### 2.5. Histopathological Examination

For histological analysis under a light microscope, the fixed ovarian tissues were first fixed in 10% neutral buffered formaldehyde and then cleaned in xylene, embedded in paraffin, sectioned at 4–6 μm thickness, and stained with hematoxylin and eosin [22]. The severity of interstitial edema, vascular dilatation, hemorrhage, and follicular degeneration were used to score the tissue structural change on a scale of 0 to 4 [23]. The categorization of follicular types was derived from the classification established by Plowchalck et al. [24]. The follicles were categorized into three stages: primordial, growing (primary and secondary), and mature follicles. A primordial follicle comprises a partial or complete layer of flattened granulosa cells around the oocyte. The primary follicle consists of an oocyte encircled by a singular layer of cuboidal granulosa cells. The secondary follicle comprises several layers of cuboidal granulosa cells encasing the oocyte, with minimal or absent antral space. A mature follicle contains a single large antral space adjacent to the oocyte.

### 2.6. Assessment of Oxidative Stress Biomarkers

Following the manufacturer’s instructions, a spectrophotometric kit (Bio-diagnostic, Cairo, Egypt) was used to quantify MDA, CAT, and SOD activities in ovarian tissue.

### 2.7. Enzyme-Linked Immunosorbent Assay (ELISA)

In accordance with the manufacturer’s instructions, ROS activities in ovarian tissue were assessed using an ELISA kit (Cat. No. MBS039665). The ELISA kit (IL-1β, SEA563Ra, 96 T; TNF-α, SEA133Ra, 96 T) was used to assess the levels of IL-1β and TNF-α in the supernatants in accordance with the manufacturer’s instructions. Rat Phosphotylinosital 3 Kinase (Pl3K) ELISA Kit (Cat. No. MBS260381), Rat AKT1 CLIA Kit (Sandwich CLIA, Cat. No. LS-F49321), and P38MAPK (Competitive ELISA, Cat. No. MBS720509) were used to test Pl3K, AKT, and P38MAPK in accordance with the manufacturer’s instructions. The levels of AMH in ovarian tissue were measured after the rat anti-Müllerian hormone (AMH) ELISA kit (Cat. No. MBS701712) was used as instructed. Serum FSH and LH levels were measured by Rat FSH, ELISA Kit Cat. No. MBS2502190, and Rat LH ELISA Kit Cat. No. MBS764675. Additionally, serum samples underwent the ELISA procedure to quantify estrogen levels utilizing the Rat Estrogen ELISA Kit (Cat No. MBS2607338).

### 2.8. RNA Extraction and Quantitative RT-PCR Analysis

Total RNA was extracted and purified from ovarian homogenates using the RNeasy micro-RNA Isolation Kit (Direct-zol RNA Miniprep Plus, Cat R2072, ZYMO RESEARCH CORP. Irvine, CA, USA). The quantity and quality of the RNA were assessed using a Beckman dual spectrophotometer (USA). Reverse transcription into cDNA was performed with the Invitrogen™ SuperScript™ IV One-Step RT-PCR System, Thermo Fisher Scientific, Waltham, MA, USA, following the manufacturer’s instructions. The relative expression levels of target genes for each sample were determined using the SuperScript IV One-Step RT-PCR kit (Cat# 12594100, Thermo Fisher Scientific, Waltham, MA, USA). Gene expression was quantified based on the threshold cycle values and normalized against a housekeeping gene, GAPDH. The control group’s relative transcript level was set to 1, allowing for comparison of relative transcript levels in other samples. The qRT-PCR primers for *TGF-β* and *Nrf2* are listed in Table 1.

### 2.9. Statistical Analysis

GraphPad Prism 9.5.1 (Graphpad Software Inc., San Diego, CA, USA) was used to conduct statistical analyses. The Shapiro–Wilk test was used to statistically examine raw data for a normal distribution. The mean ± SEM (n = 6) was used to express the data. This study employed a one-way analysis of variance (ANOVA) to evaluate the variations across the various groups. Tukey’s test for multiple comparisons was used to compare the corresponding *p* values (at a 95% confidence level) in order to evaluate significance; differences were deemed significant when *p* < 0.05.

## 3. Results

### 3.1. Histopathological Examination

**Figure 1:**
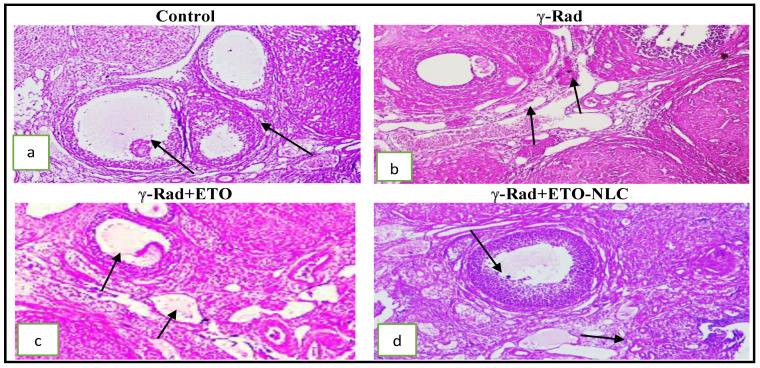
(**a**): Photomicrograph of an ovarian tissue section showing a normal architecture of ovarian cortex and medulla and different types of ovarian follicles (arrow); (**b**) photomicrograph of an ovarian tissue section showing vascular congestion, edema, and severe hemorrhage in a cortical stroma arrow; (**c**) mild congestion and edema with a thin follicular theca interna and an external arrow; (**d**) intact follicular germinal cells and mild congestion of ovarian vessels (H&E × 200).

Ovarian tissue sections of the control group showed normal architecture of the ovarian cortex and medulla with no considerable pathological changes. Different types of ovarian follicles, oocytes, and corpus luteum were seen with normal morphology. The follicular cells were arranged in a regular manner around the oocyte. Normal ovarian follicles, including primary and tertiary follicles, as well as Graafian follicles, which present with centrally located oocytes, were seen. The cuboidal germinal or follicular cells around the oocyte were arranged in a regular manner (Figure 1a). Animals in the group exposed to radiation (γ-Rad group) revealed vascular congestion edema and severe hemorrhage in the cortical stroma. There was a significant decrease in the number of primordial and primary follicles and an increase in the number of atretic follicles in this group. The corpora lutea occupies a large area in sections, as shown in Figure 1b.

On the other side, animals in the group exposed to radiation and treated by ETO (γ-Rad + ETO group) showed moderate degeneration of the ovary with some structural changes in germinal epithelium cells. Moderate congestion, edema, follicles, and thin theca interna and externa were noticed (Figure 1c). Histopathological changes were significantly mitigated in γ-Rad + ETO-NLC as compared with the radiation group. Mild degeneration of the ovary with less structural change in germinal epithelium cells and mild congestion and edema were seen. Follicles, theca interna, and externa were preserved at normal levels (Figure 1d). Also, the histological scoring and follicular score of ovarian injury in female rats was were presented in Table 2 and Table 3.

### 3.2. Oxidative Stress Markers

The effects of various treatments on MDA, CAT, and SOD levels in ovarian homogenates are summarized in Table 3. Significant variations in MDA levels were observed across the treatment groups. The control group exhibited a baseline MDA level of 0.83 ± 0.01. In contrast, the γ-Rad group showed a substantial increase in MDA levels of 493.7% (*p* < 0.0001) compared to the control. Conversely, the γ-Rad + ETO group showed a reduction in MDA to 2.89 ± 0.08 (*p* < 0.0001) compared to γ-Rad. Furthermore, the γ-Rad + ETO-NLC group displayed a further decrease in MDA levels to 1.61 ± 0.02, which was significantly lower than both the γ-Rad and γ-Rad + ETO groups (*p* < 0.0001). Also, CAT activity was significantly reduced in the γ-Rad group, with an activity level of 0.92 ± 0.006 compared to the control group’s 4.61 ± 0.09 (*p* < 0.0001). The γ-Rad + ETO group showed an increase in CAT activity to 2.07 ± 0.05 (*p* < 0.0001), while the γ-Rad + ETO-NLC group showed a further increase to 3.54 ± 0.04, significantly higher than both the γ-Rad and γ-Rad + ETO groups (*p* < 0.0001). In addition, SOD activity also displayed significant differences among the treatment groups, with the control group showing an activity level of 3.98 ± 0.03. The γ-Rad group resulted in a decline to 0.74 ± 0.01 (*p* < 0.0001). Additionally, γ-Rad + ETO increased SOD activity to 1.89 ± 0.02 (*p* < 0.0001), while the γ-Rad + ETO-NLC group exhibited an improvement to 3.00 ± 0.02, significantly higher than both the γ-Rad and γ-Rad + ETO groups (Table 4) (*p* < 0.0001).

### 3.3. Assessment of Pro-Inflammatory Cytokine Levels

**Figure 2:**
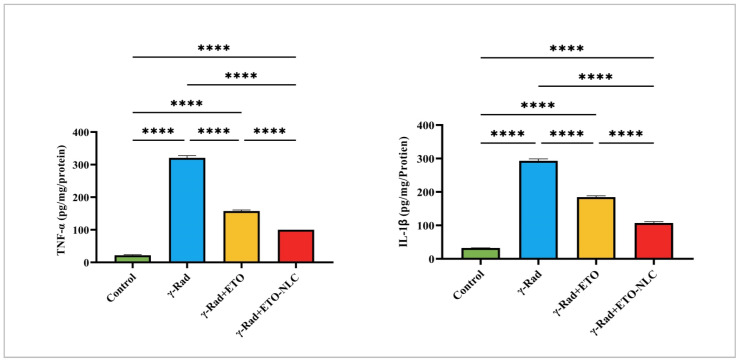
Pro-inflammatory cytokines: levels of TNF and IL-1β in γ-radiation-induced ovarian damage in female rats across experimental groups. Data were presented as mean ± SEM (n = 6). Statistical significance was assessed using ANOVA with post hoc tests, where * significant at *p* < 0.05 level; ** significant at *p* < 0.01 level; *** significant at *p* < 0.001 level; and **** significant at *p* < 0.0001 level.

The control group exhibited a mean TNF-α level of 21.70 ± 1.75, representing baseline cytokine levels. Exposure to γ-radiation resulted in a significant increase in TNF-α levels to 320.9 ± 6.67, indicating a strong pro-inflammatory response induced by radiation exposure. The treatment of γ-Rad with ETO (γ-Rad + ETO group) led to a reduction in TNF-α levels to 157.4 ± 3.37, suggesting that ETO may attenuate some of the inflammatory effects associated with γ-radiation. Further treatment with ETO-NLC (γ-Rad + ETO-NLC group) resulted in the lowest TNF-α level measured at 100.2 ± 0.15 (*p* < 0.0001), indicating a potential synergistic effect that enhances the anti-inflammatory response (Figure 2).

Similarly, the control group exhibited a mean IL-1β level of 32.51 ± 0.35, representing baseline cytokine levels. Exposure to γ-Rad resulted in a significant increase in IL-1β levels to 293.2 ± 5.59 (*p* < 0.0001), indicating a robust inflammatory response induced by radiation exposure. The γ-Rad + ETO group showed a decrease in IL-1β levels to 184.4 ± 4.06 (*p* < 0.0001), suggesting that ETO partially mitigates the inflammatory effects associated with γ-radiation. Treatment of the γ-Rad group with ETO-NLC further reduced IL-1β levels to 107.1 ± 4.05 (*p* < 0.0001), indicating that NLCs may enhance the anti-inflammatory effects of ETO. These results highlight the intricate interplay between various treatments and the modulation of TNF-α and IL-1β levels, underscoring the potential of ETO and ETO-NLC in mitigating inflammatory responses induced by γ-radiation.

### 3.4. Evaluation of AKT Activation and ROS Levels

**Figure 3:**
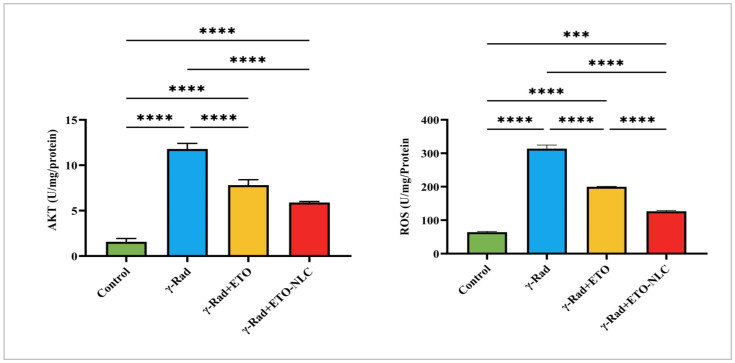
Levels of AKT and ROS in γ-radiation-induced ovarian damage in female rats across experimental groups. Data were presented as mean ± SEM (n = 6). Statistical significance was assessed using ANOVA with post hoc tests, where * significant at *p* < 0.05 level; ** significant at *p* < 0.01 level; *** significant at *p* < 0.001 level; and **** significant at *p* < 0.0001 level.

The levels of AKT and ROS were evaluated in γ-radiation-induced ovarian damage in female rats across various treatment conditions, revealing distinct patterns of response (Figure 3). The results showed that the mean AKT level was 1.563 ± 0.2092, indicating baseline activation, while the mean ROS level was 64.04 ± 2.547, representing baseline oxidative stress levels in the control group. Following γ-radiation exposure, there was a significant increase in AKT levels to 11.79 ± 0.3539, suggesting an activation of survival pathways in response to radiation exposure. Concurrently, ROS levels elevated to 313.8 ± 10.67, indicating heightened oxidative stress as a result of radiation.

The treatment of ETO led to a decrease in AKT levels to 7.804 ± 0.3455, reflecting a partial attenuation of AKT activation, while ROS levels decreased to 199.8 ± 1.467, suggesting that ETO may mitigate some oxidative stress induced by γ-radiation. Finally, the treatment of the γ-Rad group with ETO-NLC further reduced AKT levels to 5.889 ± 0.07035 and ROS levels to 126.5 ± 2.308, indicating a potential synergistic effect that enhances both signaling modulation and protection against oxidative damage.

### 3.5. Assessment of PI3K and AMPK Activities

**Figure 4:**
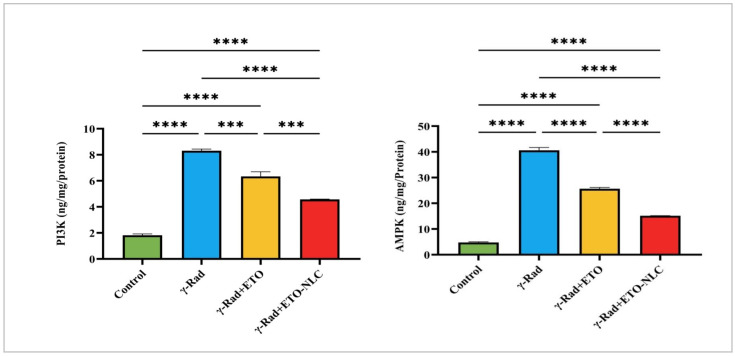
Levels of Pl3K and AMPK in γ-radiation-induced ovarian damage in female rats across experimental groups. Data were presented as mean ± SEM (n = 6). Statistical significance was assessed using ANOVA with post hoc tests, where * significant at *p* < 0.05 level; ** significant at *p* < 0.01 level; *** significant at *p* < 0.001 level; and **** significant at *p* < 0.0001 level.

Figure 4 indicates that the control group exhibited a mean PI3K level of 1.821 ± 0.1036. Following γ-radiation exposure, Pl3K levels significantly increased to 8.310 ± 0.1284, representing a 356% increase compared to the control group (*p* < 0.0001). The treatment of γ-radiation-induced ovarian damage in female rats with ETO resulted in a decrease in PI3K levels to 6.340 ± 0.3527, which corresponds to a 249% increase relative to the control (*p* < 0.0001). Further, treatment of the γ-radiation group with ETO-NLC led to a reduction in PI3K levels to 4.567 ± 0.02652, reflecting a 151% increase compared to the control (*p* < 0.0001).

Furthermore, the mean AMPK level was 4.759 ± 0.4603 in the control group. After exposure to γ-radiation, AMPK levels elevated to 40.60 ± 1.856, indicating an impressive 752% increase compared to the control (*p* < 0.0001). The treatment of ETO (γ-Rad + ETO group) resulted in a decrease in AMPK levels to 25.65 ± 0.8506, which represents a 438% increase relative to the control (*p* < 0.0001). Finally, the treatment of ETO-NLC (γ-Rad + ETO-NLC group) further reduced AMPK levels to 15.14 ± 0.04878, corresponding to a 217% increase compared to the control (*p* < 0.0001).

### 3.6. Expression Levels of TGF-β and Nrf2

**Figure 5:**
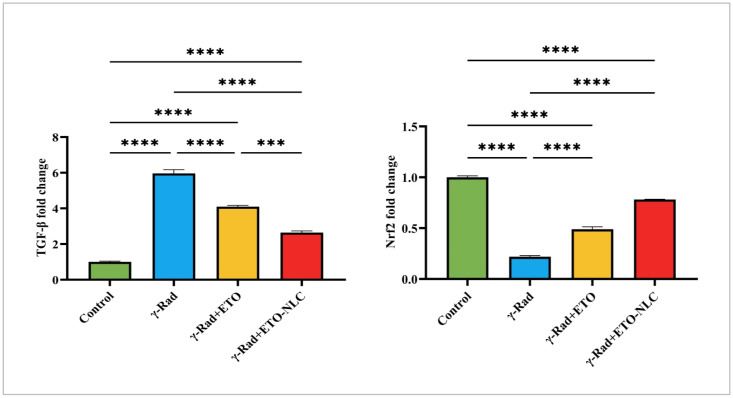
Expression levels of *Nrf2* and *TGF-β* in γ-radiation-induced ovarian damage in female rats across experimental groups. Data were presented as mean ± SEM (n = 6). Statistical significance was assessed using ANOVA with post hoc tests, where * significant at *p* < 0.05 level; ** significant at *p* < 0.01 level; *** significant at *p* < 0.001 level; and **** significant at *p* < 0.0001 level.

The expression levels of *TGF-β* and *Nrf2* were assessed across various treatment conditions, revealing distinct patterns of regulation (Figure 5). In the control group, the mean *TGF-β* level was 1.002 ± 0.04403, representing baseline expression. Following exposure to γ-radiation, *TGF-β* levels significantly increased to 5.965 ± 0.2027, indicating a robust pro-fibrotic response induced by radiation exposure (*p* < 0.0001). The treatment of ETO resulted in a decrease in *TGF-β* levels to 4.097 ± 0.07376, suggesting that ETO may partially mitigate the fibrotic effects associated with γ-radiation (*p* < 0.0001). The treatment of ETO-NLC with γ-radiation further reduced *TGF-β* levels to 2.642 ± 0.09504 (*p* < 0.0001), indicating a potential modulatory effect of the nanoparticle carrier.

Furthermore, the mean *Nrf2* level was set at 1.000 ± 0.01401 as a baseline reference for expression levels in the control group. After exposure to γ-Rad, *Nrf2* levels dramatically decreased to 0.2169 ± 0.011, indicating a significant reduction in antioxidant response signaling (*p* < 0.0001). The ETO treatment led to an increase in *Nrf2* levels to 0.4897 ± 0.02346 (*p* < 0.0001), suggesting a partial restoration of antioxidant signaling pathways. Moreover, the ETO-NLC treatment of the γ-Rad group further elevated *Nrf2* levels to 0.7818 ± 0.003129 (*p* < 0.0001), indicating enhanced activation of protective mechanisms against oxidative stress.

### 3.7. Evaluation of Hormone Levels

**Figure 6:**
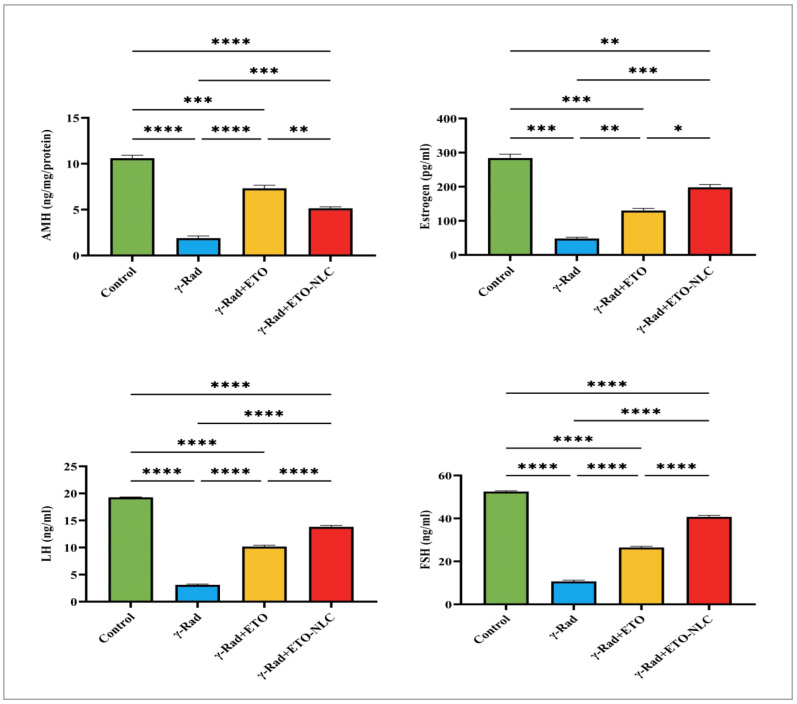
Levels of AMH, estrogen, FSH, and LH in γ-radiation-induced ovarian damage in female rats across experimental groups. Data were presented as mean ± SEM (n = 6). Statistical significance was assessed using ANOVA with post hoc tests, where * significant at *p* < 0.05 level; ** significant at *p* < 0.01 level; *** significant at *p* < 0.001 level; and **** significant at *p* < 0.0001 level.

Figure 6 indicates that the mean AMH level was 10.60 ± 0.3214 in the control group. Following exposure to γ-radiation, there was a significant decrease in AMH levels to 1.898 ± 0.2325 (*p* < 0.0001), indicating a detrimental effect on ovarian function and a substantial loss of ovarian reserve due to radiation exposure. The γ-Rad + ETO group showed an increase in AMH levels to 7.321 ± 0.3330 (*p* < 0.0001), suggesting that ETO may partially mitigate the effects of radiation on ovarian reserve. However, this level remained significantly lower than that of the control group. Furthermore, the treatment of ETO-NLC led to a further reduction in AMH levels to 5.149 ± 0.1539 (*p* < 0.001) compared to the ETO group, indicating that while ETO provides some therapeutic effects, the treatment with NLCs does not fully restore AMH levels to baseline.

The results of this study indicated changes in estrogen levels across the different treatment groups, with implications for understanding the effects of γ-radiation and subsequent treatments. The control group exhibited a mean estrogen level of 284.3 ± 11.28. In comparison, the γ-radiation group showed a dramatic decrease in mean estrogen levels to 48.10 ± 3.698, representing an 83.1% reduction from the control group, which is statistically significant (*p* < 0.001). The mean estrogen level increased to 130.0 ± 6.408 in γ-Rad + ETO, reflecting a 54.1% increase from the γ-Rad group, indicating that ETO may help mitigate some of the radiation’s detrimental effects on estrogen levels, with this change also being statistically significant (*p* < 0.01). Finally, NLC treatment resulted in a further rise in mean estrogen levels to 198.3 ± 8.091 in γ-Rad + ETO-NLC, which represents a 52.4% increase compared to the γ-Rad + ETO group (*p* < 0.05). This increase suggests that the addition of NLCs may enhance recovery further. Overall, these findings underscore the profound impact of γ-radiation on estrogen levels and highlight the potential for therapeutic strategies to restore hormonal balance following radiation exposure.

The results of this study on LH levels across different treatment groups revealed differences compared to the control group. The control group exhibited a mean LH level of 19.29. In contrast, the γ-Rad group showed a marked reduction in mean LH levels to 3.112. The γ-Rad + ETO group showed an increase in LH levels to 10.19. Additionally, the treatment of ETO-NLC led to further increases in LH levels to 13.83. The results in Figure 6 indicate that the control group exhibited a mean FSH level of 52.50. In contrast, the γ-Rad group showed a significant reduction in mean FSH levels to 10.69. The γ-Rad + ETO group showed an increase in FSH level of 26.47. Additionally, the treatment of ETO-NLC led to further increases in FSH levels to 40.71. This finding suggests that ETO and ETO-NLC may be alleviating certain detrimental hormonal effects induced by γ-radiation.

## 4. Discussion

The extensive clinical use of radiation technology for tumor identification and therapy has led to an increase in radiation exposure for patients. The ovary is highly susceptible to ionizing radiation; primordial follicles seem to be the most vulnerable of all follicle phases [25]. They cause ovarian failure by accelerating oocyte depletion through a variety of mechanisms, including oxidative damage and inflammation. Accordingly, RT increases the risk of infertility and early menopause in women by destroying and reducing ovarian follicles [26]. The female germline is found in the ovary, making it a privileged organ that is extremely vulnerable to radiation damage [27]. After surviving cancer treatments, fertile women had a decreased chance of becoming mothers [28]. This highlights the vital necessity of preserving each female cancer patient receiving RT’s reproductive health. Therefore, the current study aims to explore the potential therapeutic effects of ETO-NLC on radiation-induced ovarian damage in female rats, comparing it to conventional ETO therapy, with the goal of providing new therapeutic options.

OS primarily mediates the major mechanism of RT through ionization and the generation of ROS, which have detrimental effects on various cellular macromolecules such as membrane lipids, nucleic acids, and proteins [29]. However, radiation mostly damages the ovaries through the production of ROS, and increased ROS leads the ovaries to lose primordial follicles rapidly [30,31]. Crucially, the elevated ROS decreases female fertility and causes a fast loss of primordial follicles [31]. In the current study, ϒ-radiation reduced primordial and primary follicles and increased the number of atretic follicles in comparison to those in the control, which has an impact on the distribution of follicle sizes. The current study’s findings are in line with prior studies conducted by Mantawy et al. [32]. In addition, Haddad et al. [33] revealed that after 4 days of radiation exposure, there was a marked reduction in the size of the ovaries and a sharp decline in the populations of primordial, preantral, and antral follicles when comparing ovarian reserve indicators between control and irradiated rats. These observations were also reported by previous studies [34]. In contrast to the irradiated group, both ETO and ETO-NLC treatment resulted in less ovarian damage and led to greater preservation of various follicle populations. The current study’s results confirm that ETO-NLC effectively mitigates irradiation-induced ovarian damage, thereby leading to the preservation of various follicle populations.

IR damages cells by causing metabolic OS through the production of free radicals [35], which in turn damages macromolecules and degrades tissue function [36]. According to the current study, SOD and CAT levels decreased while upregulating MDA and ROS levels in ovarian tissues 14 days post-irradiation. The current findings are in line with earlier research that demonstrated that RT could cause endogenous antioxidants like CAT, GPX, and SOD to be depleted, which may contribute to the generation of ROS over time [37,38]. In contrast, ETO and ETO-NLC have the ability to effectively combat the harmful pro-oxidant effects of γ-radiation on ovarian tissues by downregulating ROS and MDA levels while preserving SOD and CAT activity. Consistent with these results, previous investigations indicated that ETO and ETO-NLC inhibited oxidative stress and preserved antioxidant activity in rats with radiation-induced jejunum damage [39]. Furthermore, the results of the ETO-NLC treatment demonstrated greater effectiveness compared to the ETO treatment after radiation, suggesting an enhanced activation of protective mechanisms against OS. In this way, NLCs preserve antioxidant levels within cells and shield ovarian tissue from radiation-induced injury.

*Nrf2* is considered a major OS responder, as many of its downstream target genes are included in redox imbalances in cells [40]. After being released and moving into the nucleus, *Nrf2* attaches itself to the antioxidant response element and stimulates the transcription of genes that produce antioxidants [41]. *Nrf2* mediates the elimination of ROS by targeting redox-cycling enzymes, such as SOD and CAT [7]. Furthermore, a number of investigations have illustrated the crucial function *Nrf2* plays in preventing lipid peroxidation [40]. According to Zhao et al. [42], *Nrf2* has been recognized as a crucial protective factor against IR-induced damage by facilitating DNA damage repair and antioxidant regulation. This study’s results revealed a dramatic drop in Nrf2 expression levels following radiation exposure. This suggests a major decrease in antioxidant response signaling. Conversely, both ETO and ETO-NLC treatments elevated *Nrf2* levels; additionally, ETO-NLC treatment was more efficacious than ETO, indicating better activation of defensive mechanisms against OS.

Additionally, the production of free radicals may increase pro-inflammatory pathways and activate TNF-α and IL-1β [43,44]. The current study indicates that radiation exposure raised TNF-α and IL-1β levels, suggesting a potent pro-inflammatory reaction. However, ETO and ETO-NLC treatment, initiated after irradiation, reduced TNF-α and IL-1β, underscoring their potential to mitigate inflammatory responses induced by irradiation. Moreover, the PI3K/Akt pathway, a vital survival pathway that supports cell survival, DNA repair, and anti-apoptotic signaling to prevent radiation-induced damage, is activated by a variety of signals, including TNF-α and IL-1β. The current study revealed that a 6-Gy radiation dose increased ovarian PI3K and AKT. In line with these results, a new study by He et al. [6] found that ovarian injuries caused by radiation cause oxidative damage and inflammatory injuries, lower the expression of PI3K and AKT, and cause ovarian follicular atresia. Additionally, numerous earlier studies [45,46] have indicated that oxidative and inflammatory damages activate the PI3K/Akt signaling pathway.

However, after irradiation, the ETO or ETO-NLC treatment led to a decrease in Pl3K and AKT levels; furthermore, the ETO-NLC treatment further reduced Pl3K and AKT levels, suggesting a potential synergistic effect that enhances signaling modulation against oxidative damage. Jain et al. [47] assert that ETO has repeatedly exhibited chemopreventive and anti-neoplastic properties in the initial phases of colon cancer by promoting the downregulation of the PI3K/Akt oncogenic pathway. ETO primarily inhibits the COX-2 enzyme, which synthesizes prostaglandins. These prostaglandins trigger pro-inflammatory pathways, leading to the release of TNF-α and IL-1β. Therefore, ETO can reduce the levels of these cytokines, which in turn reduces radiation damage by reducing the inflammatory response in tissues exposed to radiation. Inflammation may be decreased by ETO or ETO-NLC through modulating the PI3K/AKT signaling pathway and lowering cytokines, like TNF-α and IL-1β. Additionally, the ETO-NLC treatment had greater effects than ETO; this could be because NLCs make ETO more bioavailable, which enables a more efficient reduction in inflammatory mediators and may improve the therapeutic effect against radiation-induced damage.

Radiation-induced OS can trigger cell death by activating the apoptotic machinery that is dependent on the mitochondria. The MAPK pathway’s function in apoptotic cascades has garnered a lot of interest [48]. Moreover, the MAPK signaling pathway has recently been implicated in the death of granulosa cells caused by RT [9]. MAPK cascades are significant signaling pathways that have been linked to the regulation of ovulation and ovarian follicular development [49]. It is interesting to note that cells exposed to radiation and DNA-damaging compounds activate p38MAPK, which increases the translocation of pro-apoptotic proteins in the mitochondria and promotes apoptosis by blocking anti-apoptotic proteins [50,51]. Remarkably, the p38MAPK cascades are crucial for granulosa cell death, especially in cases of radiation-induced POF [9]. One of the main stimuli that trigger the MAPK signaling pathway is ROS [52] generated by radiation. In the current investigation, p38MAPK levels increased following radiation exposure. The data in the current study consist of previous studies by Mantawy et al. [32], which revealed that ϒ-radiation activated the MAPK pathway through considerably increased phosphorylation of p38 in ovarian follicles. Conversely, following ETO or ETO-NLC treatment, p38MAPK levels decreased. These data indicated that ETO and ETO-NLC function as inhibitors of p38MAPK activation after irradiation, potentially offering protective effects by modulating stress and inflammatory responses. The NLC formulation appears to be more effective in this role, likely due to enhanced bioavailability and drug delivery, which may improve therapeutic outcomes in radiation-related damage.

*TGF-β* is a potent pro-fibrotic factor that is increased in the early stage of radiation damage [53]. It is essential for gene expression, migration, differentiation, and proliferation; it includes three isoforms: *TGF-β1*, *TGF-β2*, and *TGF-β3* [54,55]. In the mammalian ovary, the *TGF-β* signaling pathway serves a variety of functions; it inhibits the growth of follicles’ sensitivity to FSH and controls the recruitment of primordial follicles [56]. Follicle dysplasia and ovulation failure may result from abnormally high levels of *TGF-β1* in the ovary [57]. *TGF-β* expression levels were elevated by radiation exposure in the current investigation, suggesting a strong pro-fibrotic response. ROS generated by radiation triggers *TGF-β* activation, which in turn leads to inflammation, fibrosis, and follicular depletion. According to Barcellos-Hoff and Dix [8], ROS-mediated *TGF-β* activation predominates in tissues exposed to IR. The *TGF-β* signaling system is activated by a rise in ROS following radiation exposure, which oxidizes the cysteine residues of the latency-associated peptide (LAP). When the LAP is oxidized, its conformation changes, allowing *TGF-β* to be released from the latent complex. When an active *TGF-β* binds to *TGF-β* receptors, transcription factors are phosphorylated and activated [58]. More recently, Thao et al. found that after an 8 Gy radiation exposure, the mouse glioma cell line GL261 showed increased levels of *TGF-β3 mRNA* [59]. Inversely, administering ETO or ETO-NLC to irradiated rats decreased *TGF-β mRNA* expression relative to the irradiation group. The ETO-NLC formulation seems to improve ETO distribution and efficacy, leading to a higher decrease in *TGF-β* levels in ovarian tissues post-radiation. The reduction in *TGF-β* activation by ETO-NLC provides a possible therapeutic approach to preserve ovarian function and mitigate radiation-induced ovarian damage.

Ovarian function is largely reflected in endocrine and reproductive activities. One of the most well-known endocrine indicators of ovarian reserve reduction is AMH [60,61]. The primary role of AMH is to prevent the activation of primordial follicles, which decreases the rate of ovarian reserve depletion [62]. Furthermore, AMH’s expression is minimal in atretic follicles and has been identified as a regulator of follicular atresia [63]. In clinical and experimental research, AMH was also thought to be a sensitive biomarker of iatrogenic ovarian failure [64]. AMH is a more accurate indicator of POF, according to Alipour et al. [65]. Rats exposed to radiation in this study had lower levels of AMH than the control group. Consistent with these findings, prior laboratory and human research has documented that the low or undetectable amount of AMH was assessed following irradiation exposure [66,67]. In addition, after 14 days of irradiation, serum levels of estrogen were measured, revealing that radiation reduced estrogen levels, which is consistent with prior studies [6,32]. Conversely, administering ETO or ETO-NLC to irradiated rats resulted in an increase in AMH and estrogen levels, indicating that ETO or ETO-NLC may partially mitigate the effects of radiation on ovarian reserve. Furthermore, this study’s results showed a significant decrease in FSH and LH levels after radiation exposure. The substantial decrease indicates that γ-radiation may directly inhibit the secretion of FSH and LH, potentially by impairing or modifying the function of the cells responsible for their release or by provoking a hormonal change due to radiation stress. Our findings are in accordance with Osman [68]. Conversely, the administration of ETO or ETO-NLC to irradiated rats increased LH and FSH levels. ETO and ETO-NLC may alleviate these effects by regulating inflammation and diminishing stress responses. Furthermore, ETO-NLC increased LH and FSH levels relative to ETO therapy. The NLCs may provide more accurate targeting of ETO to tissues impacted by radiation, potentially improving the drug’s capacity to restore or elevate normal hormone levels.

## 5. Conclusions

This investigation concluded that both ETO and ETO-NLC had the potential to mitigate the adverse effects of IR-induced ovarian damage by boosting antioxidant defenses and modulating oxidative stress, further suppressing the apoptotic machinery via diminishing the MAPK signaling pathway and enhancing survival pathways via the PI3K/Akt pathway, reducing fibrosis (*TGF-β*), and improving ovarian reserve, offering a promising strategy for minimizing radiation-induced ovarian damage. This study underscores the potential of ETO-NLC as an effective strategy in attenuating radiation-induced ovarian damage. These findings hold promise for future clinical applications and highlight the importance of further research in this area to enhance therapeutic interventions for preserving ovarian function in the context of cancer treatment.

## Figures and Tables

**Table 1:** Primer’s sequence of all studied genes.

	Forward Sequence	Reverse Sequence	GenBank Accession Number
***TGF-β***	GTGTGGAGCAACATGTGGAA	TTGGTTCAGCCACTGCCGTA	NC_086019.1
***Nrf2***	CCAGAAGCCACACTGACAGA	GGAGAGGATGCTGCTGAAAG	NC_086021.1
***GAPDH***	TGGATTTGGACGCATTGGTC	TTTGCACTGGTACGTGTTGAT	NM_017008.4 2

**Table 2:** Histological scoring of ovarian injury in different experimental groups.

Parameters/Groups	Control	γ-Rad	γ-Rad + ETO	γ-Rad + ETO-NLC
**Congestion**	0	3	2	1
**Edema**	0	3	2	2
**Hemorrhage**	0	4	1	0
**Follicular Degeneration**	0	3	2	1

**Table 3:** Follicular score and corpus luteum in different experimental groups.

Parameters/Groups	Control	γ-Rad	γ-Rad + ETO	γ-Rad + ETO-NLC
**Primordial Follicle**	147.7 ± 7.6	51.7 ± 7.1 ^#^	102.0 ± 10.02 ^#,$^	145.7 ± 8.8 ^$^
**Growing Follicle**	136.3 ± 2.9	46.0 ± 4.5 ^#^	102.0 ± 12.2 ^$^	148.3 ± 19.01 ^$^
**Mature Follicle**	20.0 ± 3.0	6.0 ± 1.7	15.3 ± 4.2	20.3 ± 3.2
**Corpus Luteum**	7.0 ± 2.1	15.0 ± 1.5	10.0 ± 3.7	7.4 ± 2.6

Data were represented by mean ± SEM, the value obtained from one ovary/rat (n = 3) and statistically analyzed using one-way analysis of variance (ANOVA) followed by a post hoc Tukey test. ^#^ Significant vs. the control group, ^$^ Significant vs. the radiation group at *p* < 0.05.

**Table 4:** Oxidative stress marker levels in γ-radiation-induced ovarian damage in female rats across experimental groups.

	Control	γ-Rad	γ-Rad + ETO	γ-Rad + ETO-NLC
MDA (n mol/mg/protein)	0.83 ± 0.01	4.92 ± 0.09 ^#^	2.89 ± 0.08 ^#,$^	1.61 ± 0.02 ^#,$^
CAT (U/mg/protein)	4.61 ± 0.09	0.92 ± 0.006 ^#^	2.07 ± 0.05 ^#,$^	3.54 ± 0.04 ^#,$^
SOD (U/mg/protein)	3.98 ± 0.03	0.74 ± 0.01 ^#^	1.89 ± 0.02 ^#,$^	3.00 ± 0.02 ^#,$^

Data were represented by mean ± SEM (n = 6) and statistically analyzed using one-way analysis of variance (ANOVA) followed by a post hoc Tukey test. ^#^ Significant vs. the control group, ^$^ significant vs. the radiation group at *p* < 0.05.

## Data Availability

Data will be made available upon request.
